# Data Incompleteness May form a Hard-to-Overcome Barrier to Decoding Life’s Mechanism

**DOI:** 10.3390/biology11081208

**Published:** 2022-08-12

**Authors:** Liya Kondratyeva, Irina Alekseenko, Igor Chernov, Eugene Sverdlov

**Affiliations:** 1 Shemyakin-Ovchinnikov Institute of Bioorganic Chemistry of the Russian Academy of Sciences, Moscow 117997, Russia; 2 Institute of Molecular Genetics of National Research Centre “Kurchatov Institute”, Moscow 123182, Russia; 3 Kurchatov Center for Genome Research, National Research Center “Kurchatov Institute”, Moscow 123182, Russia

**Keywords:** bioinformatics, big data, genome, systems biology, complexity

## Abstract

**Simple Summary:**

The influence of data incompleteness on the correctness of conclusions about the structure and functions of the objects under study is widely discussed in the literature. It was noted that even a small percentage of missing data can lead to incorrect conclusions and imperfect knowledge. In particular, incompleteness can lead to critical errors in the qualitative and quantitative assessments of interactions in biological systems and a distorted understanding of the functioning mechanisms of living systems. In this brief review, we attempt to demonstrate the extent of this incompleteness in functional information about living systems using the best-studied examples. We suggest that this incompleteness may form seemingly insurmountable barriers in deciphering the mechanisms of the functioning of complex systems with unpredictable properties arising from the interaction of the system components.

**Abstract:**

In this brief review, we attempt to demonstrate that the incompleteness of data, as well as the intrinsic heterogeneity of biological systems, may form very strong and possibly insurmountable barriers for researchers trying to decipher the mechanisms of the functioning of live systems. We illustrate this challenge using the two most studied organisms: *E. coli*, with 34.6% genes lacking experimental evidence of function, and *C. elegans*, with identified proteins for approximately 50% of its genes. Another striking example is an artificial unicellular entity named JCVI-syn3.0, with a minimal set of genes. A total of 31.5% of the genes of JCVI-syn3.0 cannot be ascribed a specific biological function. The human interactome mapping project identified only 5–10% of all protein interactions in humans. In addition, most of the available data are static snapshots, and it is barely possible to generate realistic models of the dynamic processes within cells. Moreover, the existing interactomes reflect the de facto interaction but not its functional result, which is an unpredictable emerging property. Perhaps the completeness of molecular data on any living organism is beyond our reach and represents an unsolvable problem in biology.

## 1. Introduction

In December 2021, a series of papers was published by a research group involved in an eight-year project to reproduce the results of cancer preclinical trials described in more than 50 papers between 2010 and 2012 [1,2,3,4]. The shockingly high irreproducibility revealed by this group has recently been discussed in various scientific journals. “One of my biggest frustrations as a scientist is that it is so hard to know which exciting results are sturdy enough to build on”; this is how Dr. Yusuf A. Hannun, director of the Stony Brook University Cancer Center in New York, reacted to the results in his comment in Nature [5]. The estimates published in other fields are quite similar. This irreproducibility calls into question the reliability of the conclusions regarding the mechanisms of disease development and, consequently, the development of methods for their treatment. Irreproducibility is not limited to cancer research. The problem was reviewed in detail by Begley and Ioannidis [6]. These authors underlined an important problem: “The variability of biological systems means that we should not expect an obligatory reproduction of the results to the smallest detail”.

Leaving aside numerous other shortcomings of the available data that have been noted, especially since the new generation of sequencing (NGS) has ushered life sciences into the era of “big data” (defined in [7,8,9,10]), the main challenge of biological big data is the low quality of the data themselves and their annotations. Genome-wide analyses give the impression of a huge amount of information, which nevertheless contains a large number of false positives and false negatives that mislead researchers [11,12,13,14].

**Figure 1:**
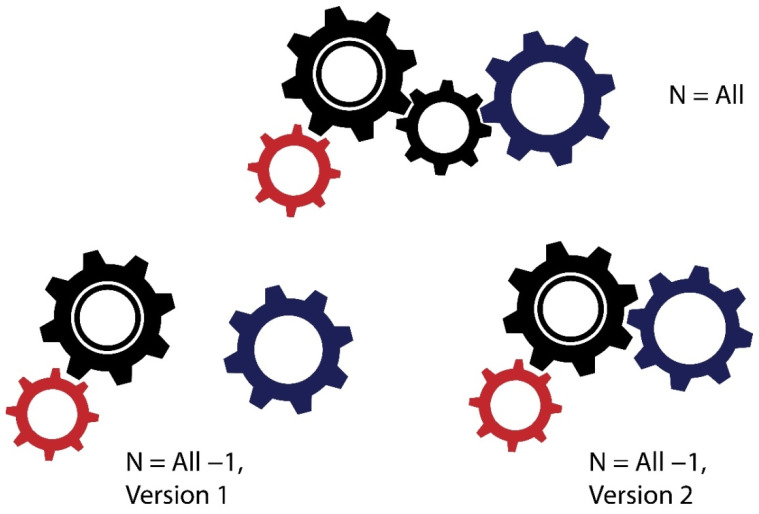
A simple illustration of the data incompleteness problem. Achieving “n = all” for many biological data may be an unreachable or unrealistic goal. With the same set of incomplete data, it is possible to arrive at different versions of the organization of any biological process.

In this review, we focus on important and seemingly insurmountable barriers faced by researchers who attempt to obtain correct conclusions about the mechanisms of life. This is data incompleteness, n ≠ all (Figure 1). To address the phenomenon of missing data, we searched the Google scholar database for review articles that include “missing data” in their title published since 2021. The search revealed 58 relevant articles. Almost all articles related to biological and medical research highlight the problem of unavoidable missing data. The problem of missing data is also widely observed in real-life databases, which often are imperfect, so that only incomplete, undefined, and invalid data sets are available. Missing data can be caused by a lot of factors, such as human errors in the experimental process, technical errors in the software or corrupt records in the databases, etc. [15].

We use for our demonstration the two most studied organisms: *E. coli*, with 600 genes—that is, 34.6% of 4623 unique genes—lacking experimental evidence of function, and *C. elegans*, with identified proteins for approximately 50% of its genes. In addition, for *C. elegans*, 23% of the protein-coding genes have no phenotypic or cell-specific expression data, and 96% of protein–protein interactions are yet to be documented. Another striking example is an artificial unicellular bacteria-like entity named JCVI-syn3.0, which has in its genome a minimal set of genes—473. In this reduced set, function could not be defined for 149 genes (31.5% of the genome). Only approximately 5–10% of all protein–protein interactions in the human interactome could be identified in recent studies [10,16,17].

These figures reflect the fundamental difficulty of deciphering the mechanisms of the functions of complex systems with unpredictable properties arising from interactions among system components.

## 2. Incompleteness of Genomic Data

Although research is currently developing in different areas within the multi-omics paradigm, the most advanced and most information-rich research is on genomes and transcriptomes and, to a much lesser extent, on proteomes. Therefore, to demonstrate the incompleteness of the available information, we mainly focus on the most advanced areas, using high-throughput NGS technologies. Interested readers can find information on multi-omics in the latest reviews [18,19,20,21]. We use two of the best-studied organisms, *E. coli* and *C. elegans*, to provide illustrative insight into the degree of data incompleteness.

## 3. *E. coli* Data Incompleteness

The first genome sequence of *Escherichia coli* was established in 1997. Meanwhile, the molecular and physiological functions of many genes are still unknown [22].

Studies on *Escherichia coli* K-12 have often been carried out with poorly annotated genes [23]. Using various databases (EcoCyc, EcoGene, UniProt, and RegulonDB), the authors identified 34.6% (1600 out of 4623) of the genes for which no function was experimentally detected. These unannotated *E. coli* genes have a name beginning with “y”, known as “y-genes”. Moreover, for 111 of the genes, the authors [23] found no information concerning their functions in the available knowledge bases such as UniProt, EcoCyc, and others. Unannotated genes in *E. coli* and other model organisms still play an important role in determining cell phenotypes [24,25,26]. The situation of the number, location, and strength of *E. coli* promoters is also incomplete. Recently (24 November 2021), 4042 promoters from the *Escherichia coli* K-12 substrain MG1655 were reported ([27], EcoCyc base, https://ecocyc.org/ (accessed on 10 December 2021)).

Only 2228 active promoters have been precisely mapped. The predicted promoter activity deduced from the genome sequence is highly unreliable [28], and the number of promoters functioning in *E. coli* is unknown, as is the extent to which the level of proteins in the cell is determined by regulation at the level of the promoter and, eventually, whether we can predict from the sequence if a promoter is contained within it, let alone the strength of the promoter and the mechanism of its regulation.

It is evident that predictions based on models of the genotype–phenotype intercommunication for whole *E. coli* cells may be highly unrealistic. In conclusion, it can be noted that in a recent study, the authors [29] compared the potential of various metrics to measure the similarity of phenotypic patterns to deduce gene function. Comparable results were obtained for most gene pairs for the three tested metrics. The conclusion was that, currently, there is no clearly preferred method of comparison.

## 4. One of the Best-Studied Multicellular Models, *C. elegans*, Is Still Very Far from “n = all”

In the late 1960s, Nobel Prize winner Sydney Brenner realized that a suitable organism for investigations into the development of the nervous system should contain a rather small number of cells, “so that exhaustive studies of lineage and patterns can be made” (quoted in [10]). The small roundworm (nematode) *Caenorhabditis elegans* was chosen for this purpose. Its relative simplicity allowed for the accumulation of data before the advent of next-generation sequencing. In an attempt to achieve “n = all” [30], numerous pieces of data were collected; the neuronal connections of all neurons were constructed from electron microscopy (302 neurons were from hermaphrodites, 385 were from males, and 294 neurons were common among them [31]), identifying the synaptic connections (“connectome”). The complete sequence of the genome, consisting of 100,291,840 base pairs, was determined [32]. 

The *C. elegans* knockout project has provided loss-of-function mutations in more than 14,000 of 20,000 protein-coding genes. It is incomplete, and it does not contain microRNAs or other RNA-modulating processes, nor control regions [10]. Recently, Li-Leger et al. [33] reported an estimation of the number of essential genes in C. elegans to be approximately 15%–30% of the 20,000 genes (for an essentiality definition see [34]). The authors identified 58 putative essential genes involved, in particular, in cell division and morphogenesis, and male-expressed genes required for fertilization and embryonic development. Another group [35] using genetic balancer systems, allowing the effective capture and maintenance of lethal mutations [36], identified 104 essential genes. This last group also reported that 604 essential genes were previously identified by other authors. One can see that the number of identified essential genes is much less than the number of previously estimated ones. More than 900 different families of transcripts have been predicted (see review in [31]). It was reported in 2015 [10] that about 10% of all TFs have been identified.

Only about 50% of the genes in *C. elegans* have been identified [10,37,38] (for comparison, about 86% of genes in humans have been associated with proteins [39], though in this case a complete standardized catalogue of protein-coding genes is also unavailable [40]). The *C. elegans* Deletion Mutant Consortium reported in 2012 the phenotypic identification of 6841 mutations in 6013 protein-coding genes [37,41]. Using Mos1 transposon insertions, 10,858 mutants in 4700 genes were exposed [42] (for a recent review, see [41]).

Identifying peptides for all exons and splice variants is still far from completion. It was assessed that 96% of the protein–protein interactions in *C. elegans* remain to be documented [43]. Such a shortage of data is also typical for other organisms. One should also keep in mind the nonlinear growth of the number of interactions with the number of proteins or genes. Therefore, “n = all” in these cases will be more difficult to achieve [44].

It was also pointed out that 23% of the protein-coding genes in WormBase release WS238 do not possess data concerning their cell-specific expression or phenotypic manifestation. There is also no GO annotation for them; therefore, they remain totally uncharacterized [10].

This lack of completeness of big data datasets poses a major challenge for data integration and the extraction of biological knowledge [10]. In addition, as we indicated before, big data collections are “noisy” and contain unreliable or even false data [10]. 

## 5. The Unsolved Mysteries of the Fully Synthetic JCVI-syn3 Genome

Recently, Venter and colleagues synthesized an artificial genome called JCVI-syn3.0, containing a minimal necessary set of DNA containing 473 genes (438 protein-coding genes and 35 genes for RNAs). The JCVI-syn3.0 genome is smaller than that of known natural independently replicating cells [45]. For 31.5% of the genes—149—of the minimized obligate genome, a specific biological function could not be ascribed [46]. Some of these important but “nonfunctional” genes appeared to be conserved across other species, including Homo sapiens. The elimination of all the “non-essential” genes resulted in a nonviable genome (reviewed in [47]). Moreover, 79 genes could not be assigned to even a broad functional category. The authors of [46] used in silico methods and assigned presumable functions to 66 of the 149 proteins. These proteins lack orthologues, lack protein domains, and/or are characteristic of membrane proteins. Among them, 24 are possible transporter proteins.

Several drawbacks, including extensive filamentation and vesicle formation, were revealed during JCVI-syn3.0 growth [48]; therefore, the new design JCVI-syn3A was constructed. It incorporated 19 additional genes from the very first version JCVI-syn1.0 that were not present in JCVI-syn3.0. With a 543 kbp genome and 492 genes (452 protein-encoding and 38 RNA-encoding), JCVI-syn3A still has a smaller genome than any natural autonomously replicating organism. However, the problems of unknown functions remained unchanged. Further information beyond the scope of this review, but which may be of interest to readers, can be found in recent articles [49,50].

In conclusion, it should be noted that missing functional annotation is quite common. It has been reported, in particular, that UniProt5 contains less than 1% of 148 million protein sequences with experimentally validated functions in gene ontology (GO) (April 2019) [46].

## 6. Complete Interactomes—An (Unreachable?) Dream of Systems Biologists

Two decades after the draft sequence of the human genome was published, the “entire” sequence was finally deciphered, with all gaps filled and errors corrected from previous versions ([51] and five accompanying papers in the same source). The complete sequence contains 3.055 billion base pairs (bps) in 22 autosomes plus Chromosome X. Nearly 200 million bps of the novel sequence were added, including 2226 paralogous gene copies; among these, 115 are probably protein-coding.

The complete reference genome sequence is very important; however, all genomic and cellular functions can be implemented only through the spatio-temporal interaction networks of cellular components [52,53,54].

Despite the advances in genome sequencing, transcriptome sequencing, and proteome sequencing, we are still far from understanding the cellular mechanisms responsible for organism function.

The entire complements of functional molecular associations that may occur in a cell are known as the “interactome”, and this encompasses a range of cellular functional networks. Complex interactome networks form through interactions among DNA and RNA molecules, also involving proteins, lipids, and small metabolites [55,56].

As a reference genome sequence is important for human genetics, reference interactome networks are critical for the full appreciation of genotype–phenotype relationships [57,58,59]. Currently, the existing information is highly fractional and represents only a small proteome fraction. Therefore, the construction and verification of the cellular interactome are two of the most important goals of genome functional analyses [60].

**Figure 2:**
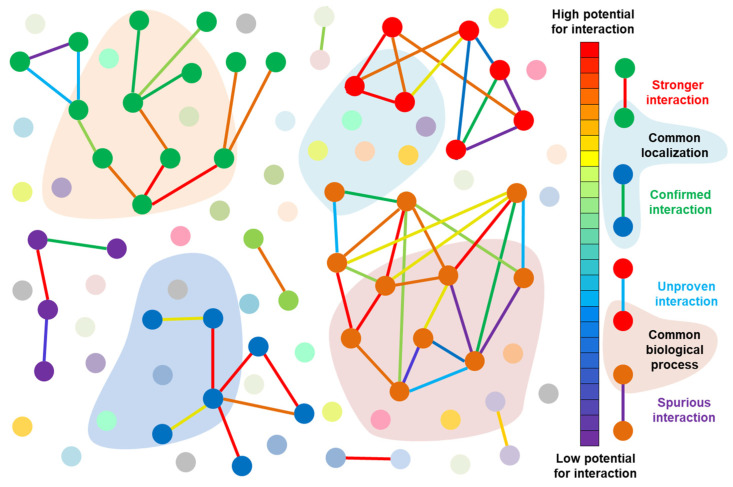
Illustration of a protein–protein interaction network: colored lines indicate an interaction between a pair of proteins, some of which are possibly spurious. The strength of the interaction between a pair of proteins is indicated by its color and is stronger when the color is closer to red. Translucent-colored clouds uniting proteins in a cluster symbolize common localization or function.

A variety of biochemical, genetic, and cellular methods have been developed for mapping interactomes [10,44,58], which are often represented as networks (graphs), allowing for both visual and computational analyses of their structure and connectivity ([61,62,63,64,65,66] with references from [67]). Figure 2 schematically illustrates the information that emerges from the research on interactomes.

Recently, “a reference map of the human binary protein interactome” was published [17]. The authors [17] indicated that it is still not possible to generate a reference map of the interactome by systematically identifying protein–protein interactions (PPIs) in thousands of cellular variations. However, various technologies [16,68] have allowed for creating maps of the human protein interactome with high functional significance. In particular, using yeast two-hybrid (Y2H) assays, the authors previously generated an interactome consisting of ~14,000 PPIs [16] and presented a reference interactome map of human binary protein interactions—the Human Reference Interactome (HuRI), containing ~53,000 high-quality PPIs, which is significantly higher than previous data. Together with genome [69], transcriptome [70], and proteome [71] data, HuRI has allowed the obtainment of more information about cellular functions. The high-quality PPI data collection can be found at http://interactome-atlas.org (accessed on 10 March 2022).

An interesting comparison demonstrating how far we are from a complete human interactome recently appeared [72]. The authors noted that since PPIs are made up of two or more proteins, the total number of PPIs is much greater than the number of human protein-coding genes (about 19000, see above), and they provided the following data on the number of PPIs reported by different sources: The Center for Cancer Systems Biology (CCSB) Interactome Database—13,993 human PPIs [16]; HuRI (see above [17])—64,006 binary interactions; the STRING database [73]—505,116 high-confidence, experimental, or predicted human PPIs. Venkatesan et al. [74] compared the PPIs found with the yeast two-hybrid method and known human PPIs, and evaluated the size of the human interactome to be ~130,000. Stumpf et al. [75] estimated the size of the interactome as 650,000. One can see significant variability in the estimation, and an understandable question appeared recently as the heading of a paper: “How Far Are We from the Completion of the Human Protein Interactome Reconstruction?” [76].

The authors [76] wrote: “After more than fifteen years from the first high-throughput experiments for human protein–protein interaction (PPI) detection, we are still wondering how close the completion of the genome-scale human PPI network reconstruction is, what needs to be further explored, and whether the biological insights gained from the holistic investigation of the current network are valid and useful”. They suggest that “an almost complete picture of a structurally defined network has been reached”. 

However, practically all major processes in a cell are implemented not by binary PPI interactions but by complexes consisting of several participants, and the cell can be considered an interactome of protein machines [77,78].

As indicated by Alberts [77], the cell can be thought of as a factory containing a complex network of interconnected assembly lines of large molecular machines. In addition, these machines are in permanent motion, whereas our data on interactomes are inevitably static [77]. Alberts also indicated that although we have undoubtedly made great progress in deciphering the structure of protein assemblies, we still have an enormous amount left to learn. Understanding a protein machine function will require, along with knowledge of its static structure, knowledge of the kinetics and energetics of all the intermediate products that are formed during the reaction. This remains a problem that will require methodologies that do not yet exist. The question is whether it will ever be possible to develop, for example, a technique that allows researchers “to follow the kinetics and structure of each of the intermediates involved in the many fascinating transport reactions that occur deep within the lipid bilayer membrane”. Alberts wrote about this in 1998; almost a quarter of a century has passed, but the situation has not changed considerably (see, for example [79,80,81]).

In the meantime, intensive work continues to attempt to use the capabilities of modern computer tools for the analysis of available interactomes, especially bacterial ones. As illustrative examples, one can cite the studies of Wuchty et al. [82] and Dilucca et al. [83].

In the first study [82], the authors attempted to summarize and analyze protein interactions using data from different sources. Two main observations stand out: (i) the evolutionary conservation of some interactions and the complete absence of others. For example, 80 protein complexes of *H. pylori* were also observed in *Escherichia coli*, whereas 120 complexes were not found in *E. coli*; (ii) the comparison of various species allowed for the obtainment of the putative functions of approximately 300 poorly characterized or previously uncharacterized proteins. 

In the second study [83], the authors related the evolutionary conservation, essentiality, and functional repertoire of a gene to the connectivity k (the number of interprotein links) in the PPI network of 42 bacteria with genomes of different sizes, and reasonably separated evolutionary trajectories. In particular, they demonstrated that highly connected proteins (with connectivity a k ≥ 40) were encoded by genes that were conserved and essential among the species considered [84]. Despite the undoubtedly interesting results, they should be treated with reasonable caution because of the incomplete nature of the interactomes used for the analyses. Huxley’s (Thomas H. Huxley, the famous English biologist and anthropologist) warning always stands: “Mathematics may be compared to a mill of exquisite workmanship, which grinds you stuff of any degree of fineness; but, nevertheless, what you get out depends upon what you put in; and as the grandest mill in the world will not extract wheat flour from peas cods, so pages of formulae will not get a definite result out of loose data” [85].

## 7. Bio-Databases and Ontologies for Biomedical Literature: The Inherent Incompleteness of Gene Ontology

Thousands of global biodatabases currently exist (reviewed in [86]). UniProtKB now includes more than nine million entries. Swiss-Prot annotates UniProtKB records, and it is expected that it will soon contain 500,000 protein sequences. The research team manually annotated approximately half of these entries by analyzing thousands of articles and data from hundreds of other databases. The team members have processed an enormous amount of information: an estimated 25,000 peer-reviewed journals publishing about 2.5 million articles per year. For life sciences alone, this amounts to two new articles published in MEDLINE each minute [86].

We will use just one of the databases, gene ontology (GO), as an illustrative example. GO is a universal portal for operations with high-capacity biological datasets [87]. It is a formidable resource and it is relatively easy to use without a deep understanding of its structure. GO is very useful when dealing with large databases and data mining; however, as we explain later, we have to treat this information with caution. A very widespread type of analysis involves comparing gene sets using their functional annotations to detect the enrichment or depletion of functional groups in a given sample of genes. It can also be used to determine the relationship of certain functions to regulatory networks, sequence convergence or divergence, and other aspects of gene activities and evolution. Since the appearance of its introductory article [88], about 280,000 papers with the words “gene ontology” have been published (Google Scholar). 

The following can be found in the current release (1 July 2022): 43,558 GO terms, 7,483,496 annotations, 1,480,259 gene products, and 5213 species (http://geneontology.org/, accessed on 10 July 2022). However, most of its gene annotations are scanty and incomplete [89,90] (also see below). As a result, it is rather difficult to identify associations among genes and a huge number of terms. The ontology is very dynamic and constantly improving in order to better represent the evolutionary and functional relationships among organisms. However, numerous aspects of the gene ontology database are still poorly comprehended. One should keep in mind that the information contained in the GO database is necessarily incomplete (see below for more details), and the absence of functional evidence does not mean that there is no function [87]. This is described as the open world assumption. Disregarding the open world assumption can cause inflated large numbers of false positive rates when using gene function prediction tools. Numerous pitfalls, depending on the structure of GO, the methods of compiling annotations, and their variability based on newly emerging data (dictating the need to use the latest version of GO), are given in two brilliant reviews [88,89].

As noted by Gaudet regarding GO, the central database for functional genetics, there are “misconceptions and misleading assumptions commonly made about GO, including the effect of data incompleteness, the importance of annotation qualifiers, and the transitivity or lack thereof associated with different ontology relations” [87]. 

While great efforts are being made to increase the coverage of annotated gene products, it should not be expected that eventually every gene product will be annotated. 

Another problem is that the incompleteness is very uneven. In addition, even rather well-annotated GO parts can also cause trouble, providing users with seemingly contradictory results.

## 8. Conclusions: Is Data Completeness One More Unsolvable Problem of Biology?

The famous scientist Jan Baptista van Helmont (1580–1644) undertook an experiment to demonstrate where trees obtain material from for growth. He grew a willow with a pre-weighed volume of soil. After five years, he found that the willow weighed about 74 kg more than at the beginning. Since the weight of the soil did not change much, van Helmont concluded that the additional plant material only came from the water. Van Helmont was unaware of photosynthesis. This incomplete knowledge led to incorrect conclusions. This is a useful lesson for both data producers and analysts.

The incomplete nature of the available data is exacerbated by the fact that we do not really know how far we are from completeness. Although attempts are being made with the help of computer analysis to predict completeness (see, for example, [79,80,81]), it is not clear how realistic these predictions are given the poor quality of the data on which they are based.

In addition, there is a strongly uneven distribution among organisms, among their tissues and cells, and among their compartments [91]. Some of the reasons for this unevenness are objective, for example, the difficult accessibility of certain cells and tissues, while others are often of an organizational nature. This last problem was discussed by Alberts [91], who named it the “Canalization of Research Areas on the Principle of Training Inertia”. He noted that while many important areas of cell biology are little explored, there are overcrowded areas of science where many scientists conduct almost identical experiments. This is most likely because numerous students of each major scientist, after receiving a doctoral degree, create their own laboratories where they continue research in the same area. This may be because researchers are concerned about the risk of entering a new area, whereas the completeness of scientific knowledge requires many people to work in unexplored areas. Alberts gives the example of *Escherichia coli*, for which a little more than 4000 different proteins have been detected. However, even today, we have no idea what ~1000 of them do (see above). This is an unexplored area in which almost no one works today. This situation also occurs in more complex organisms. However, if we have such gaps in our knowledge, we cannot hope to understand the mechanisms of functioning of even the simplest living cells, not to mention human cells [92]. The modern system of rewards in science (the publication of articles and distribution of grants) supports this fear. Therefore, we must consider ways to encourage research in risky areas. Interestingly, when Alberts and others were making their fundamental discoveries, this fear was not so prevalent. An outstanding example is the work of Brenner on the development of the nematode *Caenorhabditis elegans* as an object of study in molecular genetics. Brenner decided to study this transparent worm to solve an important new problem in molecular biology, and he started this around 1966. The first paper on genetics and a number of *C. elegans* mutants, written exclusively by Brenner, appeared in 1974 [93]. For eight years, he worked without a single publication. Today, it is impossible to imagine the effectiveness of a scientist not being evaluated by the number of articles published.

Among the objective reasons for data incompleteness, the two most important are (i) the complex nature of the interactions among biological systems components, and (ii) that the majority of processes in cells are dynamic and occur over a large range of time scales. Both of these are discussed above. 

In summary, every major process in a cell is materialized by complexes consisting of tens of protein molecules, each of which interacts with several other large complexes of proteins often connected to membranes or nucleic acids [77,78]. Combining many components with nonlinear interactions leads to new emergent properties that can only be understood in the context of the whole system. The emergent properties are nonlinear and cannot be described by the linear superposition of the experimental data [94,95].

Most of the available data are static snapshots, and it is barely possible to generate realistic models of the dynamic processes within cells occurring in times from milliseconds to years [94].

As a result, as formulated by Sydney Brenner: “We are drowning in a sea of data and starving for knowledge. Biological sciences have exploded largely through our unprecedented power to accumulate descriptive facts. How to understand genomes and how to use them is going to be a central task of our research in the future” [96]. In other places, Brenner asked: “Is there some other approach? If I knew it, I would be doing it, and not writing about the problem” [92].

We believe that no one knows. Some time ago, one of us indicated three unsolvable problems in biology: “It is impossible to create two identical organisms, to defeat cancer, or to map organisms onto their genomes” [95]. Perhaps the unattainability of the completeness of molecular data for any living organism is the fourth unsolvable problem.

Finally, it should be noted that old discussions of whether a “hypothesis first” [97] or “data first” [98] acceptance of the data incompleteness throw additional weight on the “hypothesis first” side, provided, of course, that the completeness and consistency of the data underlying the hypothesis are good enough (for a more current discussion, see: [99,100]). Completeness (the proportion of stored data against the potential of “n = all”) is one of the most important characteristics of big data quality [101,102]. Another important characteristic is “consistency”; consistent representation is the degree, in particular, to which data are compatible with previous data [103]—that is, reproducible.

We have attempted to demonstrate that biological data (especially in the case of cancer) are incomplete and may be poorly reproducible. It seems axiomatic that incomplete data cannot be the basis for constructing a correct theory or for designing an effective treatment system in medicine. This has recently been dramatically demonstrated by a series of hasty and incorrect conclusions drawn from incomplete data on the effectiveness of anti-infection drugs against SARS-CoV-2 [104]. However, as the history of science shows, even incomplete data can serve as a basis for proposing and testing hypotheses, which must be consistent with the fundamental principle of falsifying (discrediting), that is, containing a system of its own refutation. The principle was put forward in 1934 by Sir Karl Popper [105], and since then has proven its significance time and again. This was once again confirmed by the previously mentioned Taran et al. [104] on the example of SARS-CoV-2 drug searches.

Unfortunately, we do not know what we do not know. This important problem was discussed in a comprehensive review by Hutter and Moerman [10]. The authors noted that it is difficult to even define completeness, that achieving n = all for many biological data may be an unreachable or unrealistic goal, and that now the problem is whether there is a point at which data collection is “good enough,” even if it is not comprehensive. Depending on the scientific question, it might be possible to obtain a sufficiently “complete” understanding of a complex system functioning with a limited data set. They believe that “not being able to achieve n = all does not necessarily mean that a scientific question is unsolvable”. We completely agree with such an optimistic point of view.

The general strategies for data completeness improvement and minimization of the incompleteness effect were reviewed in detail by Begley and Ioannidis [6] and Danchin et al. [14]. Are there any strategies for the improvement of data completeness? At the dawn of genomics in 2000, Nature Genetics published a commentary titled “Grass-roots genomics” where the authors gave the following recipe for deciphering the organisms: “Genomic technologies can give hints about the functions of genes, but the information they get is usually too limited to draw any conclusions from it. These hints can only point the way to discovery if they are used by someone with experience and intuition to see the direction they point. There is only one way—gene by gene, process by process, researcher by researcher—by which we can “decipher the organism” [106].

## Data Availability

Not applicable.
